# Monolayer-to-bilayer transformation of silicenes and their structural analysis

**DOI:** 10.1038/ncomms10657

**Published:** 2016-02-05

**Authors:** Ritsuko Yaokawa, Tetsu Ohsuna, Tetsuya Morishita, Yuichiro Hayasaka, Michelle J. S. Spencer, Hideyuki Nakano

**Affiliations:** 1 TOYOTA Central R&D Labs, Inc., 41-1, Yokomichi, Nagakute, Aichi 480-1192, Japan; 2 CD-FMat, National Institute of Advanced Industrial Science and Technology (AIST), Central 2, 1-1-1 Umezono, Tsukuba, Ibaraki 305-8568, Japan; 3 The Electron Microscopy Center, Tohoku University, Katahira 2-1-1, Aoba-ku, Sendai 980-8577, Japan; 4 School of Science, RMIT University, GPO Box 2476, Melbourne, Victoria 3001, Australia; 5 JST Presto, Kawaguchi 332-0012, Japan

## Abstract

Silicene, a two-dimensional honeycomb network of silicon atoms like graphene, holds great potential as a key material in the next generation of electronics; however, its use in more demanding applications is prevented because of its instability under ambient conditions. Here we report three types of bilayer silicenes that form after treating calcium-intercalated monolayer silicene (CaSi_2_) with a BF_4_^−^ -based ionic liquid. The bilayer silicenes that are obtained are sandwiched between planar crystals of CaF_2_ and/or CaSi_2_, with one of the bilayer silicenes being a new allotrope of silicon, containing four-, five- and six-membered *sp*^3^ silicon rings. The number of unsaturated silicon bonds in the structure is reduced compared with monolayer silicene. Additionally, the bandgap opens to 1.08 eV and is indirect; this is in contrast to monolayer silicene which is a zero-gap semiconductor.

## Abstract (web summary)

Two dimensional forms of silicon offer different conductive properties to that of the bulk material, promising applications in new electronic technologies. Here, the authors report the fabrication of bilayer silicenes which, unlike their monolayer form, are indirect bandgap semiconductors.

## Graphical abstract

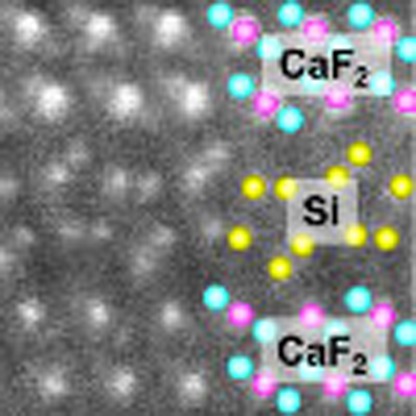

A frenzy of interest in graphene has spawned many theoretical and experimental studies^1^,^2^,^3^,^4^. After calculating the structures of two-dimensional (2D) crystals of silicon (silicene)^5^,^6^,^7^, researchers have speculated that silicon atoms might form graphene-like sheets and have attempted to produce such silicene structures^8^,^9^,^10^,^11^,^12^. Very recently, Tao *et al*.^13^ succeeded in fabricating the first silicene transistor, although the device's performance was modest. Nonetheless, the development of much more facile and practical processing methods has remained a challenging issue. The most difficult problem is that silicene grows on specific substrates and is stable only under vacuum conditions^8^,^9^,^14^,^15^. Another issue is that the influence of the substrate cannot be removed; the strong hybridization between Si and the substrate may stabilize silicene grown on specific substrates^8^,^14^,^15^,^16^.

In a previous report on calcium-intercalated silicene (CaSi_2_), we observed a massless Dirac-cone band dispersion at the k-point in the Brillouin zone, which was located far from the Fermi level because of the substantial charge transfer from the Ca atoms to the silicene layers^17^. This result is similar to the previously reported band structures of silicenes deposited on specific substrates^9^ because CaSi_2_ is a type of Zintl silicide, in which the formal charge is rewritten as Ca^2+^ and Si^−^ (ref. 18). Therefore, the intrinsic electronic structure of silicene has never been observed. In the calculated results, a van der Waals bonded silicene layer has been deposited on an intact multi-CaF_2_ layer^19^. If the Ca layer of CaSi_2_ had been exchanged with a CaF_2_ layer, the influence of the substrate would have been almost completely suppressed. To reduce the influence of external factors on the electronic structure of silicene (for example, from substrates or counter ions) and to increase the stability under ambient condition, we replaced monolayer silicene with bilayer silicene.

The existence of a bilayer silicene structure, whose density of unsaturated silicon bonds is reduced in comparison with monolayer silicene, has been predicted by molecular dynamics (MD) calculations^20^,^21^,^22^,^23^,^24^,^25^,^26^,^27^. If we could experimentally prepare a similar bilayer silicene, we could then investigate its intrinsic electronic structure. Because of the electron transfer from the calcium cation, the monolayer silicene in CaSi_2_ is a formally anionic layer^17^: when the calcium cation becomes electrically neutral, the silicene will not retain its honeycomb structure and will reconstruct to form a more stable structure. Under this supposition, we attempted to segregate the Ca and Si phases while maintaining the layer structures by diffusing fluoride (F) atoms, which are more electronegative than Si, into CaSi_2_; the goal was to form an ionic bond (or interaction) between Ca and F. In this study, BF_4_ anion based ionic liquid was used for the origin of fluoride anion.

## Results

### Fluoride diffusion into CaSi_2_

**Figure 1:**
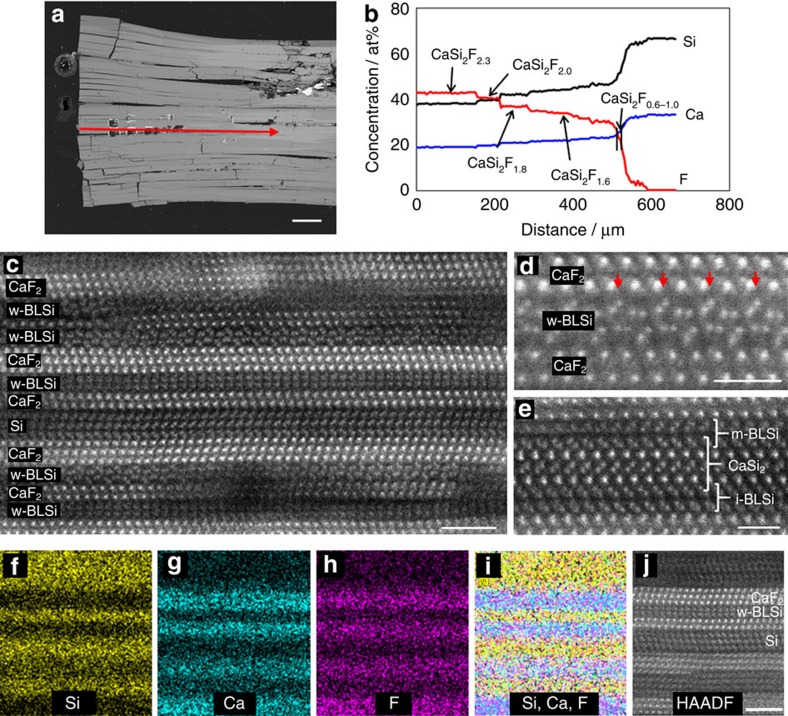
Visualization of fluoride diffusion. (**a**) Cross-sectional BSE image of the crystal grain including CaSi_2_F_X_ compound. (**b**) EPMA quantitative line analysis result along the red arrow in **a**. (**c**) HAADF-STEM image taken from a region with CaSi_2_F_1.8_ in **b**; the strip contrast corresponds to Si (dark domain) and CaF_2_ (bright domain) planar crystals. (**d**) An enlarged HAADF-STEM image taken from a region with CaSi_2_F_2_ in **b**; red arrows indicate an F-vacancy site. (**e**) HAADF-STEM image taken from a region with CaSi_2_F_0.6-1.0_ in **b**; bright dots, corresponding to the projected atomic positions of m-and i-BLSi, can be observed in the image. (**f**–**i**) STEM-EDX elemental mapping results of the CaSi_2_F_2_ composition region. One-element mapping (**f**: Si; **g**: Ca; and **h**: F). (**i**) Overlapped-mapping of Si, Ca and F. (**j**) HAADF-STEM image of the STEM-EDX elemental mapping area. The scale bars in **a**; **c** and **j**; and **d** and **e**; 100 μm, 2 nm and 1 nm.

When the CaSi_2_ crystal (Supplementary Fig. 1) was annealed in [BMIM][BF_4_] ionic liquid at 250–300 °C, it was changed to a CaSi_2_F_X_ (0≤X≤2.3) compound through diffusion of F^−^, in which the local F^−^ concentration gradually decreased from the crystal edge to the interior (Fig. 1a,b and Supplementary Fig. 2). As a result, three types of bilayer Si in a CaSi_2_ single crystal were obtained by diffusion of F^−^. Figure 1c, which displays a high-angle annular dark field scanning transmission electron microscopy (HAADF-STEM) image taken of the CaSi_2_F_1.8_ compound, shows the alternate stacking of planar crystal domains with layer thicknesses of 1–2 nm. The HAADF-STEM imaging provided an atomic-scale *Z*-contrast image (*Z*: atomic number) to distinguish the heavier constituent elements^28^,^29^,^30^. STEM-energy-dispersive X-ray spectroscopy (STEM-EDX) elemental mapping identified the bright-contrast crystal domains, which were identified as the CaF_2_ phase and the dark domains, which were identified as Si phases (Fig. 1f–j). We determined the crystal structures of the entire planar region in the images of the CaSi_2_F_1.8_ and CaSi_2_F_2.0_ compounds shown in Fig. 1c,d, respectively. These planar domains were identified as trilayer CaF_2_, trilayer Si, bilayer CaF_2_ and a novel bilayer silicene (denoted as w-BLSi in Fig. 1c,d) that has not been previously predicted by MD calculations^20^,^21^,^22^,^23^,^24^,^25^,^26^,^27^. Furthermore, two types of bilayer silicenes, one with inversion symmetry (i-BLSi) and one with mirror symmetry (m-BLSi), were recognized in the CaSi_2_F_0.6-1.0_ composition area (Fig. 1e and Supplementary Fig. 3). The formation of m-BLSi is in accordance with predictions from a previous MD study^22^. The i- and m-BLSi must be adjacent to a pair of CaF_2_ and CaSi_2_ crystal layers. The abundance ratio of i-BLSi to m-BLSi was 124:3 in the observed HAADF-STEM images. Because the calculated energy of i-BLSi was 0.03 eV per atom lower than that of m-BLSi under vacuum, the abundance ratio is qualitatively reasonable. The average size of w-BLSi is ∼30 nm, and that of m-BLSi is ∼10 nm. The size of i-BLSi is greater than 51 nm, which is the maximum size that can be observed by STEM imaging.

### Structural determination of w-BLSi

**Figure 2:**
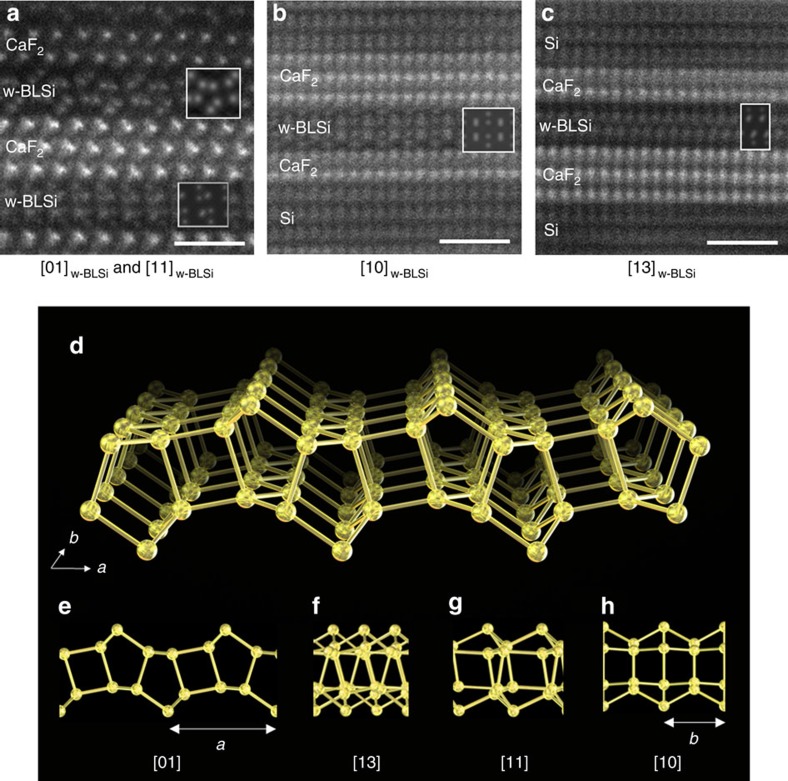
Structural determination. (**a**–**c**) HAADF-STEM and simulation (insets) images of w-BLSi. (**a**) the [01]_w-BLSi_ and [11]_w-BLSi_ incident directions ([1-10]_CaF2_), (**b**) the [10]_w-BLSi_ and [11-2]_Si and CaF2_ directions and (**c**) the [13]_w-BLSi_ and [11-2]_Si and CaF2_ directions. (**d**) Schematic illustration of the w-BLSi atomic structure. (**e**–**h**) Schematic structures projected in each direction in **e** [01], **f** [13], **g** [11] and **h** [10] directions. All scale bars in (**a**–**c**), 1 nm.

The atomic structure of the bilayer silicene was determined from HAADF-STEM images that were taken with different incident electron beam directions (Fig. 2a–c, Supplementary Fig. 4 and Supplementary Note 1). As shown in Fig. 2d, the bilayer silicene structure had a 2D translation symmetry and a wavy morphology (hereafter, we refer to the structure as w-BLSi). The w-BLSi structure consists of two silicenes, with alternating chair and boat conformations, that are vertically connected via four-, five- and six-membered rings. Because w-BLSi consists of only Si atoms exhibiting tetrahedral coordination, the top atom of the five-membered silicon ring possesses unsaturated silicon bonds (dangling bonds). Therefore, compared with those in monolayer silicene and i- (or m-) BLSi, the density of unsaturated silicon bonds in w-BLSi decreased to 25 and 50%, respectively (Supplementary Fig. 5).

We determined the atomic positions of w-BLSi from high-resolution transmission electron microscopy and HAADF-STEM images as accurately as possible (Supplementary Figs 6 and 7, Supplementary Table 1 and Supplementary Note 2). The 2D translation periods of w-BLSi were *a*=0.661(2) nm and *b*=0.382(3) nm, and the two translation axes were normal to each other (Supplementary Fig. 8). The *a* period of w-BLSi is similar to the triple lattice spacing of d_11-2_ in CaF_2_ (0.223 nm), and the *b* period is similar to d_-110_ in CaF_2_ (0.386 nm); that is, the difference between w-BLSi and CaF_2_ (111) is less than the observation error (Supplementary Fig. 9). Because the atomic arrangement of the (111) plane of the CaF_2_ crystal exhibited threefold symmetry, three equivalent relative rotation angles were observed between w-BLSi and the CaF_2_ (111) plane (Supplementary Figs 10 and 11). In addition, the angle between the [01]_w-BLSi_ and [11]_w-BLSi_ directions was almost 60° (Supplementary Fig. 10, w-BLSi is described in 2D notation, because 2D can be expressed more simply than three dimensions). Therefore, Figs 1c and 2a show the contrast of two different arrangements of bright dots—specifically, the [01] and [11] direction images (Fig. 2e,g) in the w-BLSi regions. In almost all of the observed HAADF-STEM images, w-BLSi always faced the (111) plane of CaF_2_, and the F vacancies (red arrows in Fig. 1d) on the CaF_2_ (111) surface were recognized at special positions associated with the wavy structure of w-BLSi. A w-BLSi was observed to be sandwiched between two CaF_2_ layers with an F-site surface vacancy of ∼0.5 at the interface (Fig. 1d, Supplementary Figs 7,12–15 and Supplementary Note 3).

### DFT and *ab initio* MD calculations and optical properties

**Figure 3:**
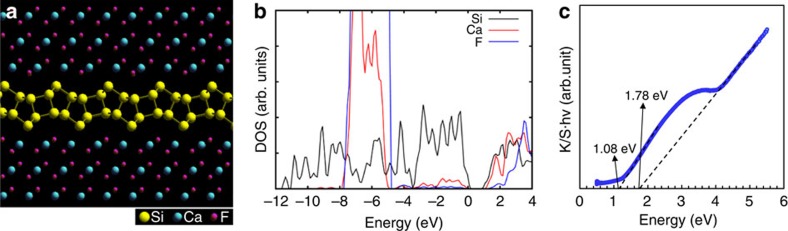
DFT and *ab initio* MD results and optical property. (**a**) Structure of w-BLSi sandwiched between two CaF_2_ crystals, with vacancies at half of the F sites on the interface; this structure was used to calculate the DOS and was obtained from the transformation of i-BLSi in the *ab initio* MD simulation and the subsequent quenching process (Supplementary Method). (**b**) Decomposed DOS for Si, Ca and F in w-BLSi displayed in **a**. (**c**) Plot of multiplication of the K/S and energy as a function of energy for CaSi_2_F_1.8-2.3_ consisting of w-BLSi, trilayer silicene with dangling bonds and F-terminated trilayer silicene. The absorption spectrum suggests two indirect gaps with values of 1.08 and 1.78 eV.

The w-BLSi structure appears to resemble re-BLSi^20^ in appearance; however, its atomic arrangement is clearly different (Supplementary Fig. 16). An *ab initio* MD calculation was performed for BLSi under the conditions corresponding to the experimentally observed structure, that is, BLSi was sandwiched between two CaF_2_ layers with an F-site surface vacancy of 0.5 at the interface. The MD calculation was started with the i-BLSi structure, but it was immediately transformed to another BLSi structure. The system was then equilibrated, and the resultant BLSi structure was found to perfectly agree with the experimentally observed w-BLSi structure in Fig. 3a (Supplementary Tables 2–5, Supplementary Fig. 17 and Supplementary Note 4). The electronic density of states (DOS) for w-BLSi was calculated by using the structure in Fig. 3a, and the decomposed DOSs for Si, Ca, and F are shown in Fig. 3b. The Ca and F bands are located far below the Fermi level, and the valence bands consist of only Si bands. An ionic rather than a covalent interaction is thus expected between Si and Ca or F. We also observe that the bandgap opens to ∼0.65 eV, in contrast to monolayer silicene, which is a zero-gap semiconductor^31^. Interestingly, however, the gap closes when w-BLSi is isolated without geometry optimization under vacuum (Supplementary Fig. 18b). This result indicates that, in the CaSi_2_F_X_ compound, charge transfer from Ca to Si occurs, filling the energy levels that are unoccupied under vacuum (Supplementary Discussion). Thus, the electronic properties of w-BLSi appear to be sensitive to its environmental conditions.

The presence of the F vacancies allows the electrons on Ca to transfer to Si, which enhances the stability of the w-BLSi structure (Fig. 3a) by saturating the dangling bonds. The CaF_2-X_ domains (specifically, ionic crystalline domains) surrounding the Si layers are key to the formation of the w-BLSi structure.

The optical bandgap can be calculated from the absorption spectrum. The diffuse reflectance spectrum of the powder sample with CaSi_2_F_1.8-2.3_ composition was measured, and the obtained reflectance spectrum data (Supplementary Fig. 19) were converted to a Kubelka–Munk function (K/S), which is proportional to the absorption coefficient (α). The sample was a mixture of w-BLSi, two types of trilayer silicene (with dangling bonds and terminated with F atoms, as shown in Supplementary Fig. 20) and a CaF_2_ layer (Supplementary Note 5). The relationship between the absorption coefficient (α) and the bandgap energy (Eg) can be described by two types of equations: αhν=const (direct gap) and αhν=A (hν−Eg) (indirect gap), where the DOS for 2D crystals is constant as a function of energy^32^,^33^,^34^,^35^ (Supplementary Note 5). Here, h, ν and A are Planck's constant, light frequency and proportional constant, respectively. From two linear fittings of the spectrum, the latter equation was found to be suitable for the sample. The absorption edges of the CaSi_2_F_1.8-2.3_ compound were observed at 1.08 and 1.78 eV (Fig. 3c), assuming indirect transitions.

Freestanding trilayer silicene is semi-metallic, as shown by density functional theory (DFT) calculations^36^. It has been suggested that the bandgap of trilayer silicene with dangling bonds in CaSi_2_F_1.8-2.3_ is nearly zero if charge transfer between the trilayer silicene and the CaF_2_ layer is inhibited^19^. From previous DFT results of monolayer and multilayer silicene terminated with atoms^37^,^38^, it is conjectured that the bandgap of F-terminated trilayer silicene would be ∼1 eV within the framework of the DFT and Perdew, Burke and Ernzerhof (PBE) technique. It should be noted that DFT calculations using a standard generalized gradient approximation functional tend to underestimate the bandgap (roughly ∼2/3 in crystal Si). This indicates that the bandgap experimentally measured for the trilayer silicene should be ∼1.5 eV. Meanwhile, the bandgap for w-BLSi, which is estimated to be ∼ 0.65 eV in the DFT–PBE calculation, is expected to be ∼1 eV in the experimental measurement. Therefore, the measured gaps were estimated such that the gaps of w-BLSi and F-terminated trilayer silicene were 1.08 and 1.78 eV, respectively.

### Transformation process from monolayer silicene to w-BLSi

**Figure 4:**
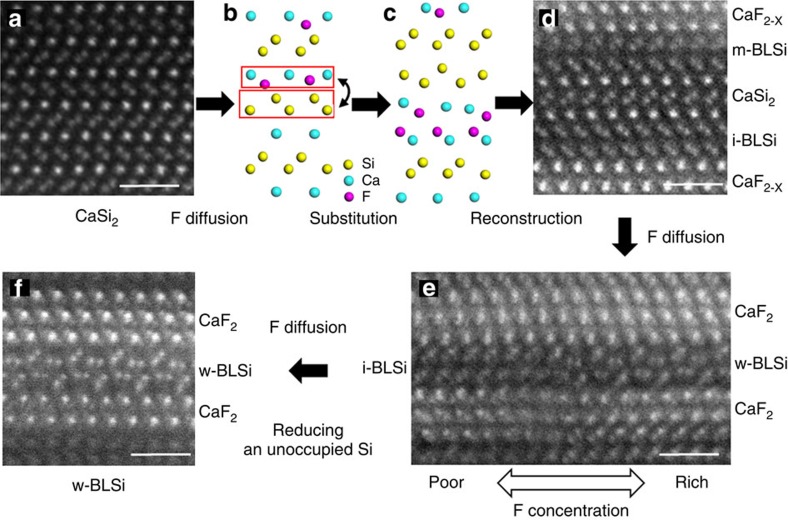
A model for the transformation process from monolayer Si to w-BLSi. (**a**) and (**d**–**f**) HAADF-STEM image. (**b**,**c**) A schematic model. (**a**) Raw tr6 CaSi_2_. (**b**) F diffusion into CaSi_2_. (**c**) A random arrangement of i-BLSi and bilayer CaF_2-X_ in CaSi_2_. (**d**) i-BLSi, CaF_2-X_ and CaSi_2_ in a region with CaSi_2_F_0.6-1.0_. (**e**) i-BLSi and w-BLSi formed within the same layers in CaSi_2_F_0.6-1.0_. (**f**) w-BLSi in CaSi_2_F_2.0_. All scale bars, 1 nm.

On the basis of the HAADF-STEM data, we discussed a model for the transformation process from a monolayer silicene in CaSi_2_ (Fig. 4a) to w-BLSi (Fig. 4f). When F^−^ ions diffuse from the surface of a CaSi_2_ crystallite into the crystal along the Ca layer, thin CaF_2-x_ planar crystals are formed; as a result, anionic silicene layers assemble to reduce the number of unsaturated bonds beyond the Ca layer (Fig. 4b). During this movement, the Si covalent bonding network with honeycomb symmetry is broken and its arrangement consequently becomes random (Fig. 4c). As shown in Fig. 4d, two types of bilayer silicenes, i-BLSi and m-BLSi, which formed in the slit-like regions, as predicted by the MD calculation^22^, co-exist with CaSi_2_ in the low F-concentration region. Both of these structures are stabilized as a result of charge transferred from the Ca atoms which saturate the silicon dangling bonds.

We analysed more than 200 STEM images of BLSi; w-BLSi was recognized at F concentrations surpassing that of CaSi_2_F_1.8_. With increasing F concentration, the site occupancy of F concentration at the interface of the CaF_2_ planar crystal reached ∼0.5, then w-BLSi was formed by the change of ionic interactions among Si, Ca and F (Fig. 3e,f). In this process, negatively charged Si atoms tend to lose their electrons, which makes i- (or m-) BLSi less stable because the ‘capping' of the dangling bonds by extra electrons from Ca is reduced and the dangling bonds destabilize the *sp*^3^ tetrahedral configuration. Thus, the anionic honeycomb structure of i- (or m-) BLSi is transformed to w-BLSi, which is approximately neutral because of the fluorination of the Ca cation.

## Discussion

We focused on calcium-intercalated silicene (CaSi_2_) and discovered a strategy for transforming monolayer silicene into a novel bilayer silicene (w-BLSi). From HAADF-STEM images, we observed that w-BLSi was formed between the planar crystals of CaF_2_ and contained four-, five- and six-membered silicon rings, although w-BLSi consists of only Si atoms exhibiting tetrahedral coordination. Compared with monolayer silicene, the number of unsaturated silicon bonds in w-BLSi decreased to 25% of the unit cell. The transformation process from monolayer silicene in CaSi_2_ to w-BLSi was estimated from HAADF-STEM data. When F^−^ ions diffuse into the CaSi_2_ crystal along the Ca layer, thin CaF_2-x_ planar crystals and two types of bilayer silicenes (i-BLSi and m-BLSi) are formed, following breakage of the Si covalent bonding monolayer network. Both of these Si structures were stabilized as a result of charge transferred from the Ca atoms which saturate the silicon dangling bonds. With increasing F content, i- (or m-) BLSi is transformed to w-BLSi. Additionally, the structure possesses an indirect bandgap of 1.08 eV in contrast to monolayer silicene, which is a zero-gap semiconductor.

## Methods

### Synthesis of CaSi_2_F_X_ compound

CaSi_2_ single-crystal grains (0.1 g) were reacted with 5 ml of ionic liquid [BMIM][BF_4_] (1-butyl-3-methylimidazolium tetrafluoroborate) at 300 °C for 15 h. BF_4_^−^ decomposed into F^−^ during annealing, and the CaSi_2_ crystal was changed to CaSi_2_F_X_ compounds (0≤X≤2.3) through the diffusion of F^−^ (Fig. 1a,b). More details are given in Supplementary Method.

### Chemical composition analysis

The chemical compositions of the CaSi_2_F_X_ domains were determined by electron probe microanalyser (EPMA) with a wave dispersion system (JEOL JXA-8200), an accelerating voltage of 10 kV, a specimen current of 50 nA, and an electron irradiation area of 5 μmϕ. Single-phase CaF_2_ and Si crystals were used as the standard for quantitative composition analysis of Ca, F and Si. EPMA line analyses were performed with 5 μm steps from the edge to the inside of the CaSi_2_F_X_ crystallites cross-sectioned parallel to the CaSi_2_ [001] direction.

### TEM/STEM analysis

HAADF-STEM observations^28^,^29^,^30^ and STEM energy-dispersive X-ray spectroscopy (EDX) analyses were performed with a Titan^3^. G2 60–300 electron microscope (FEI, Cs=156 nm) operated at 300 kV. HAADF-STEM imaging was capable of providing an atomic-scale Z-contrast image associated with the heavier constituent elements. The annular detector was set to collect the electrons scattered at angles between 50.5 and 200 mrad. High-resolution transmission electron microscopy observations were obtained with a JEM-2000EX electron microscope (JEOL, Cs=0.7 mm) operating at 200 kV. TEM specimens of CaSi_2_F_X_ were detected with five different F concentration ranges (CaSi_2_F_0.6_-_1.0_, CaSi_2_F_1.6,_ CaSi_2_F_1.8_, CaSi_2_F_2.0_ and CaSi_2_F_2.3_) by using the FIB micro-sampling method^39^. The atomic positions in the w-BLSi crystal and the interface structure were characterized by comparing the HAADF-STEM image contrasts with simulated contrasts calculated by the multi-slice method using MacTempasX.

### Computational method

DFT and *ab initio* MD calculations were performed to calculate the DOS and to examine the structural stability of BLSi using the Vienna *Ab initio* Simulation Package (ref. 40). The projector augmented wave method^41^ and generalized gradient approximation with the exchange and correlation functions of PBE were employed^42^. A plane-wave basis set with an energy cutoff of 400 eV was used with Γ-point sampling in the Brillouin zone. To model the BLSi systems observed in our experiments, two-layer Si structures were sandwiched by CaF_2_ crystal domains, each consisting of three sets of CaF_2_ layers, with or without the F-site vacancy at the Si/CaF_2_ interfaces. The DOS for the w-BLSi was calculated for the structure obtained after the quenching process (shown in Fig. 3a) following the 300 K run. More details are given in Supplementary Method.

### Optical reflectivity

Diffuse reflectance spectra were obtained for the CaSi_2_F_1.8-2.3_ composition powder sample using a spectrophotometer (JASCO V-670).

The diffuse reflectance spectra were processed under the Kubelka–Munk formalism, and the bandgaps were determined using a plot of the multiplication of the K/S and energy. More details are given in Supplementary Methods.

## Additional information

**How to cite this article:** Yaokawa, R. *et al*. Monolayer-to-bilayer transformation of silicenes and their structural analysis. *Nat. Commun.* 7:10657 doi: 10.1038/ncomms10657 (2016).

## Supplementary Material

Supplementary Information Supplementary Figures 1-20, Supplementary Tables 1-5, Supplementary Notes 1-5, Supplementary Discussion, Supplementary Methods and Supplementary References

## References

[b1] Novoselov K. S. . Electric field effect in atomically thin carbon films. Science 306, 666–669 (2004). 15499015 10.1126/science.1102896

[b2] Novoselov K. S. . Two-dimensional gas of massless dirac fermions in graphene. Nature 438, 197–200 (2005). 16281030 10.1038/nature04233

[b3] Zhang Y., Tan Y. W., Stormer H. L. & Kim P. Experimental observation of the quantum hall effect and berry's phase in graphene. Nature 438, 201–204 (2005). 16281031 10.1038/nature04235

[b4] Novoselov K. S. . Room-temperature quantum Hall Effect in Graphene. Science 315, 1379 (2007). 17303717 10.1126/science.1137201

[b5] Takeda K. & Shiraishi K. Theoretical possibility of stage corrugation in Si and Ge analogs of graphite. Phy. Rev. B 50, 14916 (1994). 10.1103/physrevb.50.14916 9975837

[b6] Cahangirov S., Topsakal M., Aktürk E., Şahin H. & Ciraci S. Two- and one-dimensional honeycomb structures of silicon and germanium. Phys. Rev. Lett. 102, 236804 (2009). 19658958 10.1103/PhysRevLett.102.236804

[b7] Lebègue S. & Eriksson O. Electronic structure of two-dimensional crystals from *ab initio* theory. Phys. Rev. B 79, 115409 (2009).

[b8] Vogt P. . Silicene: Compelling experimental evidence for graphenelike two-dimensional silicon. Phys. Rev. Lett. 108, 155501 (2012). 22587265 10.1103/PhysRevLett.108.155501

[b9] Fleurence A. . Experimental evidence for epitaxial silicene on diboride thin films. Phys. Rev. Lett. 108, 245501 (2012). 23004288 10.1103/PhysRevLett.108.245501

[b10] Okamoto H. . Silicon nanosheets and their self-assembled regular stacking structure. J. Am. Chem. Soc. 132, 2710–2718 (2010). 20121277 10.1021/ja908827z

[b11] Sugiyama Y. . Synthesis and optical properties of monolayer organosilicon nanosheets. J. Am. Chem. Soc. 132, 5946–5947 (2010). 20387885 10.1021/ja100919d

[b12] Okamoto H., Sugiyama Y. & Nakano H. Synthesis and modification of silicon nanosheets and other silicon nanomaterials. Chem. Eur. J. 17, 9864–9887 (2011). 21780200 10.1002/chem.201100641

[b13] Tao L. . Silicene field-effect transistors operating at room temperature. Nat. Nanotechnol. 10, 227–231 (2015). 25643256 10.1038/nnano.2014.325

[b14] Morishita T., Spencer M. J. S., Kawamoto S. & Snook I. K. A new surface and structure for silicene: polygonal silicene formation on the Al(111) surface. J. Phys. Chem. C 117, 22142–22148 (2013).

[b15] Gao J. & Zhao J. Initial geometries, interaction mechanism and high stability of silicene on Ag(111) surface. Sci. Rep. 2, 861 (2012). 23155482 10.1038/srep00861 PMC3498736

[b16] Cahangirov S. . Electronic structure of silicene on Ag(111): strong hybridization effects. Phys. Rev. B 88, 035432 (2013).

[b17] Noguchi E. . Direct observation of dirac cone in multilayer silicene intercalation compound CaSi_2_. Adv. Mater. 27, 856–860 (2015). 25502913 10.1002/adma.201403077

[b18] Yaokawa R., Nakano H. & Ohashi M. Growth of CaSi_2_ single phase polycrystalline ingots using the phase relationship between CaSi_2_ and associated phases. Acta Mater. 81, 41–49 (2014).

[b19] Kokott S., Pflugradt P., Matthes L & Bechstedt F. Nonmetallic substrates for growth of silicene: an *ab initio* prediction. J. Phys. Condens. Matter 26, 185002 (2014). 24728001 10.1088/0953-8984/26/18/185002

[b20] Morishita T., Spencer M. J. S., Russo S. P., Snook I. K. & Mikami M. Surface reconstruction of ultrathin silicon nanosheets. Chem. Phys. Lett. 506, 221–225 (2011).

[b21] Sakai Y. & Oshiyama A. Structural stability and energy-gap modulation through atomic protrusion in freestanding bilayer silicene. Phy. Rev. B 91, 201405(R) (2015).

[b22] Morishita T., Nishio K. & Mikami M. Formation of single- and double-layer silicon in slit pores. Phy. Rev. B 77, 081401(R) (2008).

[b23] Bai J., Tanaka H. & Zeng X. C. Graphene-like bilayer hexagonal silicon polymorph. Nano Res 3, 694–700 (2010).

[b24] Johnston J. C., Phippen S. & Molinero V. A single-component silicon quasicrystal. J. Phys. Chem. Lett. 2, 384–388 (2011).

[b25] Pflugradt P., Matthes L. & Bechstedt F. Unexpected symmetry and AA stacking of bilayer silicene on Ag(111). Phys. Rev. B 89, 205428 (2014).

[b26] Guo Z.-X. & Oshiyama A. Structural tristability and deep Dirac states in bilayer silicene on Ag(111) surfaces. Phys. Rev. B 89, 155418 (2014).

[b27] CahangirovS. . Atomic structure of the 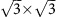 phase of silicene on Ag(111). Phys. Rev. B 90, 035448 (2014).

[b28] Pennycook S. J. & Jesson D. E. High-resolution incoherent imaging of crystals. Phys. Rev. Lett 64, 938–941 (1990). 10042119 10.1103/PhysRevLett.64.938

[b29] Pennycook S. J. & Jesson D. E. High-resolution z-contrast imaging of crystals. Ultromicroscopy 37, 14–38 (1991).

[b30] Pennycook S. J. & Jesson D. E. Atomic resolution Z-contrast imaging of interfaces. Acta Mater. 40, S149–S159 (1992).

[b31] Huang S., Kang W. & Yang L. Electronic structure and quasiparticle bandgap of silicene structures. Appl. Phys. Lett. 102, 133106 (2013).

[b32] Lee P. A., Said G., Davis R. & Lim T. H. On the optical properties of some layer compounds. J. Phys. Chem. Solids 30, 2719–2729 (1969).

[b33] Mak K. F., Lee C., Hone J., Shan J. & Heinz T. F. Atomically thin MoS_2_: a new direct-gap semiconductor. Phys. Rev. Lett. 105, 136805 (2010). 21230799 10.1103/PhysRevLett.105.136805

[b34] Gaiser C. . Band-gap engineering with HfS_X_Se_2√X_. Phys. Rev. B 69, 075205 (2004).

[b35] Bianco E. . Stability and exfoliation of germanane: a germanium graphane analogue. ACS Nano 7, 4414–4421 (2013). 23506286 10.1021/nn4009406

[b36] Kamal C., Chakrabarti A., Banerjee A. & Deb S. K. Silicene beyond mono-layers —different stacking configurations and their properties. J. Phys. Condens. Matter 25, 085508 (2013). 23370369 10.1088/0953-8984/25/8/085508

[b37] Gao N., Zheng W. T. & Jiang Q. Density functional theory calculations for two-dimensional silicene with halogen functionalization. Phys. Chem. Chem. Phys. 14, 257–261 (2012). 22083171 10.1039/c1cp22719j

[b38] Morishita T. . First-principles study of structural and electronics properties of ultrathin silicon nanosheets. Phys. Rev. B 82, 045419 (2010).

[b39] Kirk E. C. G., Williams D. A. & Ahmed H. Cross-sectional transmission electron microscopy of precisely selected regions from semiconductor devices. Inst. Phys. Conf. Ser. 100, 501–506 (1989).

[b40] Kresse G. & Furthmüller J. Efficiency of *ab-initio* total energy calculations for metals and semiconductors using a plane-wave basis set. Comput. Mater. Sci. 6, 15–50 (1996). 10.1103/physrevb.54.11169 9984901

[b41] Blöchl P. E. Projector augmented-wave method. Phys. Rev. B 50, 17953 (1994). 10.1103/physrevb.50.17953 9976227

[b42] Perdew J. P., Burke K. & Ernzerhof M. Generalized gradient approximation made simple. Phys. Rev. Lett. 77, 3865 (1996). 10062328 10.1103/PhysRevLett.77.3865

