# Supplementary material for: Monolayer-to-bilayer transformation of silicenes and their structural analysis
Source: Nat Commun. 2016 Feb 5;7:10657. doi: 10.1038/ncomms10657 (PMC4748253; doi:10.1038/ncomms10657)
Supplement: Supplementary Information — Supplementary Figures 1-20, Supplementary Tables 1-5, Supplementary Notes 1-5, Supplementary Discussion, Supplementary Methods and Supplementary References [file ncomms10657-s1.pdf]

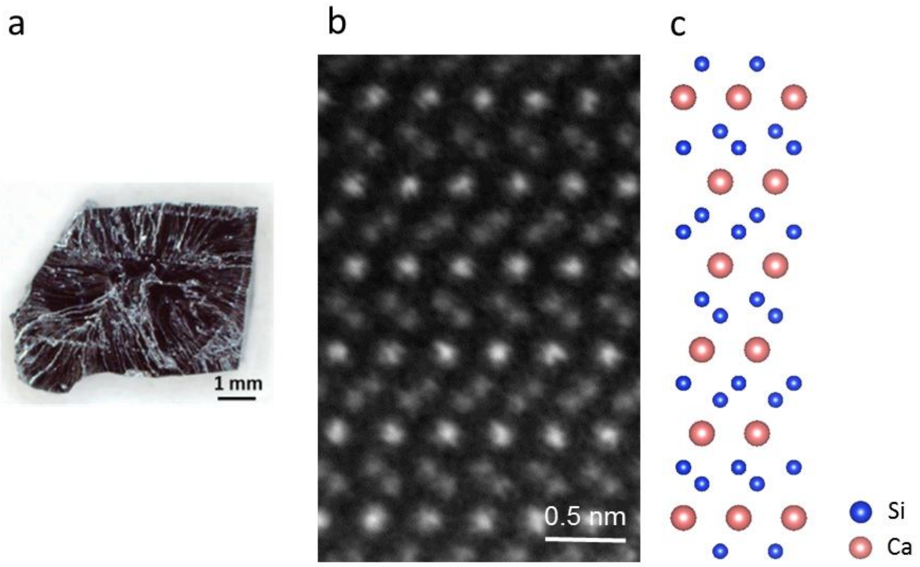

Supplementary Figure 1 | a, $CaSi_2$ single crystal. **b**, HAADF-STEM image of [100] incidence. **c**, A predicted structure of tr6-$CaSi_2$.

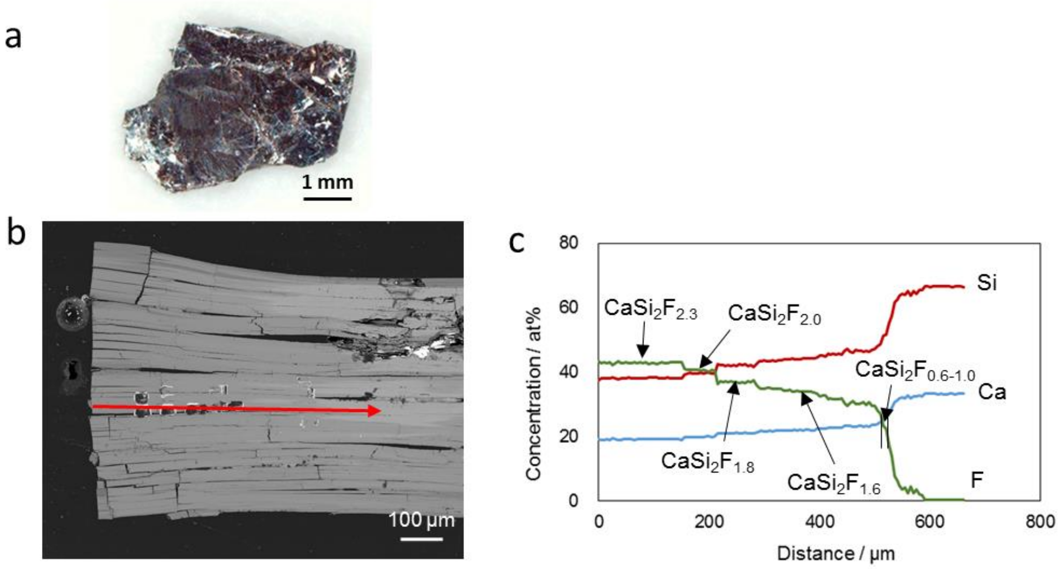

Supplementary Figure 2 | a, $CaSi_2F_X$ compound single crystal. **b**, Cross-sectional BSE image of the crystal grain including $CaSi_2F_X$ compound. **c**, EPMA quantitative line analysis result along the red arrow in Supplementary Fig. 2b. The surface of the $CaSi_2F_X$ compound particle shows a dark color caused by the adhesion of degraded

ionic liquid, whereas the interior of the particle features a metallic luster. The F concentration gradually decreased from the edge to the interior in the cross-section of both $CaSi_2F_X$ compound particle. The $CaSi_2$ crystal was changed to a $CaSi_2F_X$ compound ($0 \leqq x \leqq 2.3$), through the diffusion of F^-. The Si:Ca concentration ratio was kept constant at 2:1 in the entire area of both compounds. The F concentration of the $CaSi_2F_X$ compound indicated three constant (plateau) composition regions ($CaSi_2F_{1.8}$, $CaSi_2F_{2.0}$, $CaSi_2F_{2.3}$); however, the plateau is not always observed in $CaSi_2F_X$ compound particles. The ionic liquid [BMIM][BF_4] used for annealing consists of H, B, C, N and F. Because the B, C and N concentrations of the $CaSi_2F_X$ compound are below 0.1 wt%, which is within the detection limit, only F is recognised in the $CaSi_2F_X$ compound; we note that it is not possible to detect H by EPMA.

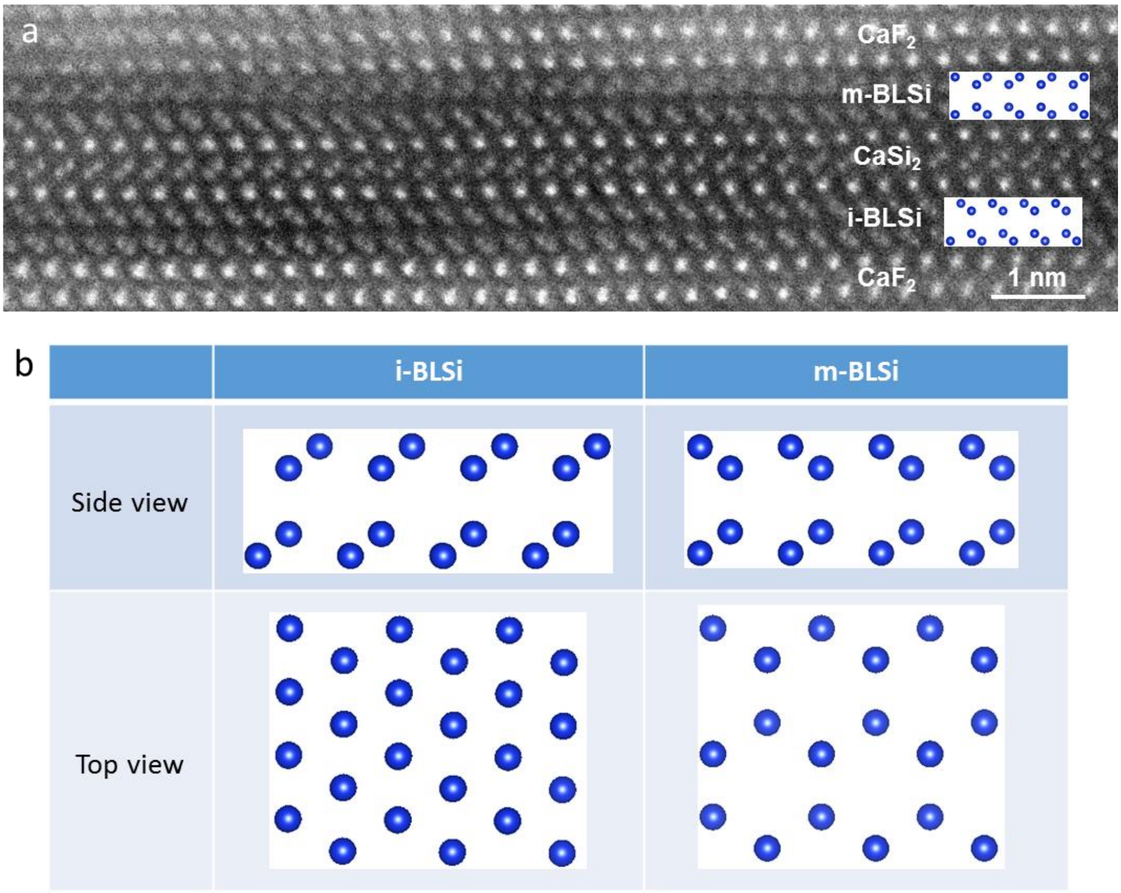

Supplementary Figure 3 | a, HAADF-STEM image of the $CaSi_2F_{0.6\text{-}1.0}$ composition area. i- and m-BLSi models are inserted. **b**, Models of i- and m-BLSi.

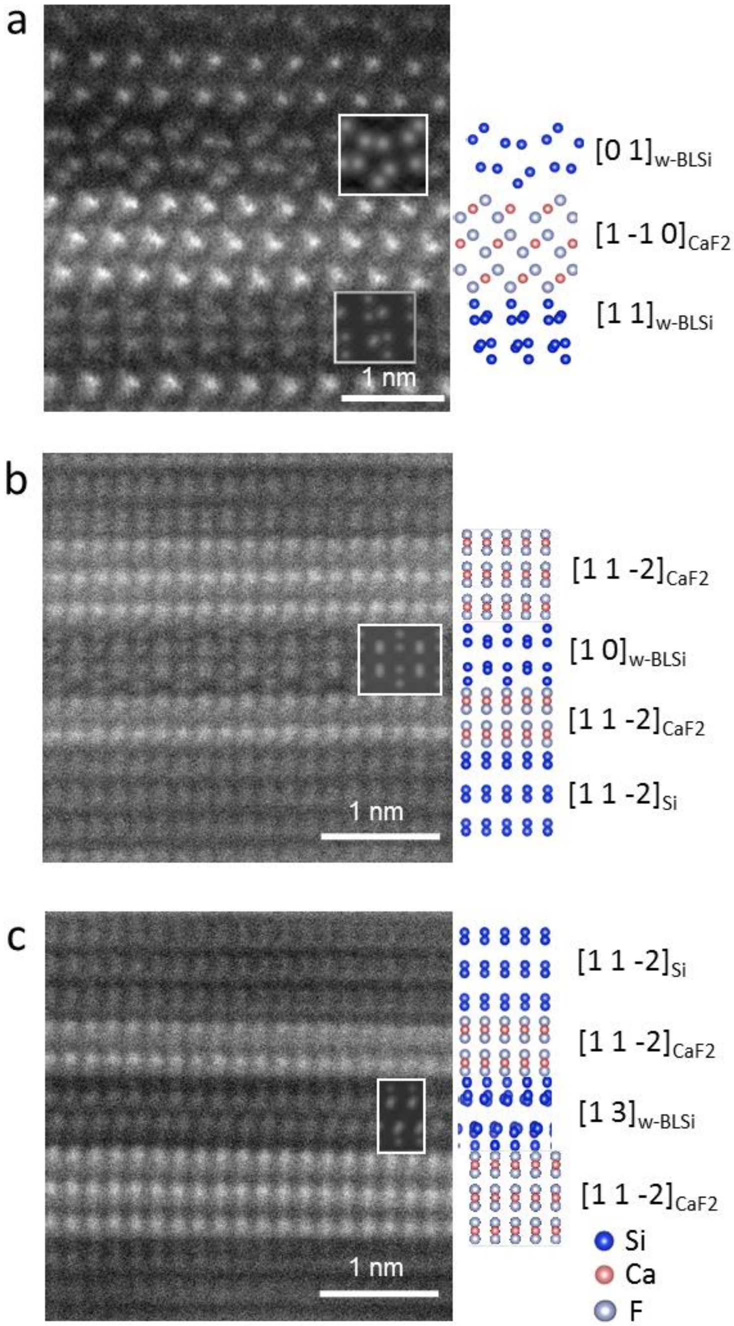

Supplementary Figure 4 | HAADF-STEM images and simulations of w-BLSi in four directions and projected models. The simulation images of w-BLSi are inserted in the HAADF-STEM image. **a**, HAADF-STEM image in the $[01]_{w\text{-}BLSi}$ and $[11]_{w\text{-}BLSi}$ incident directions ($[1\text{-}10]_{CaF2}$). **b**, HAADF-STEM image in the $[10]_{w\text{-}BLSi}$ and $[11\text{-}2]_{Si\ and\ CaF2}$ directions. **c**, HAADF-STEM image in the $[13]_{w\text{-}BLSi}$ and $[11\text{-}2]_{Si\ and\ CaF2}$ directions.

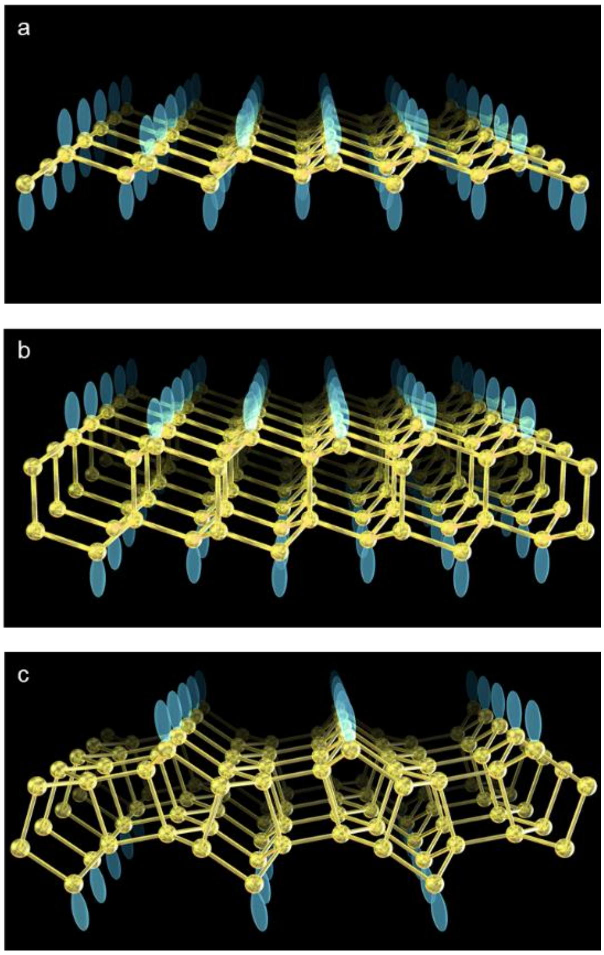

Supplementary Figure 5 | Schematics of the free-standing atomic structures of monolayer and bilayer silicene. **a**, Monolayer silicene; **b**, m-BLSi; **c**, w-BLSi. Unsaturated Silicon bond images are shown by blue color parts.

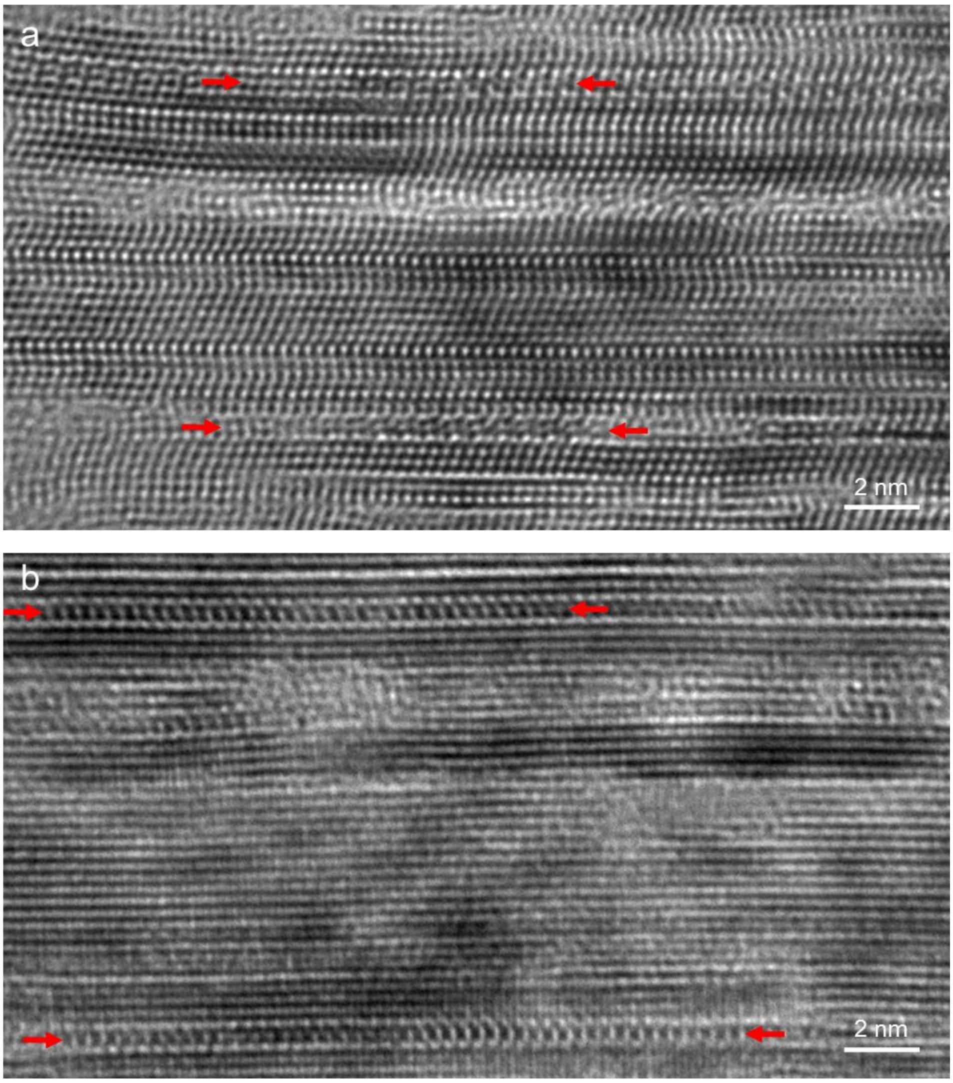

Supplementary Figure 6 | a, HRTEM image in the $[01]_{w\text{-}BLSi}$ incident direction ($[1\text{-}10]_{Si\ and\ CaF2}$); **b**, HRTEM image in the $[10]_{w\text{-}BLSi}$ ($[11\text{-}2]_{Si\ and\ CaF2}$) incident direction. The red arrows indicate the w-BLSi positions.

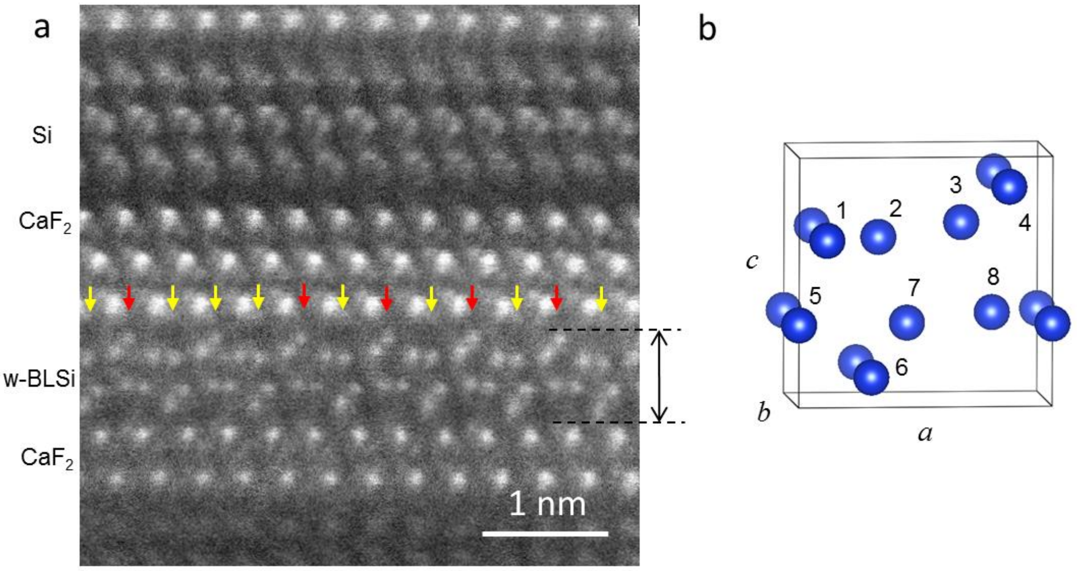

Supplementary Figure 7 | a, [-1 1 0]$_{\mathrm{Si\ and\ CaF2}}$ and [0 1]$_{\mathrm{w\text{-}BLSi}}$ incident direction HAADF-STEM image of Si, CaF_2 and the w-BLSi formed area. F atoms (yellow arrows) and vacancies (red arrows) almost alternately substitute for F sites at the interface between w-BLSi and CaF_2. **b**, Schematic unit cell of the three-dimensional w-BLSi crystal.

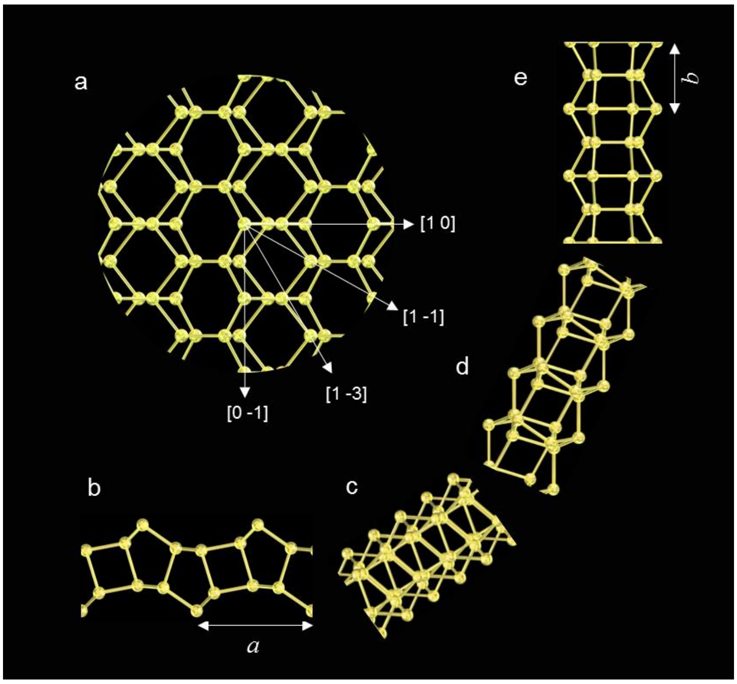

Supplementary Figure 8 | Schematic structures projected in each direction. **a**, Stacking direction. **b**, [01] direction. **c**, [13] direction. **d**, [11] direction. **e**, [10] direction.

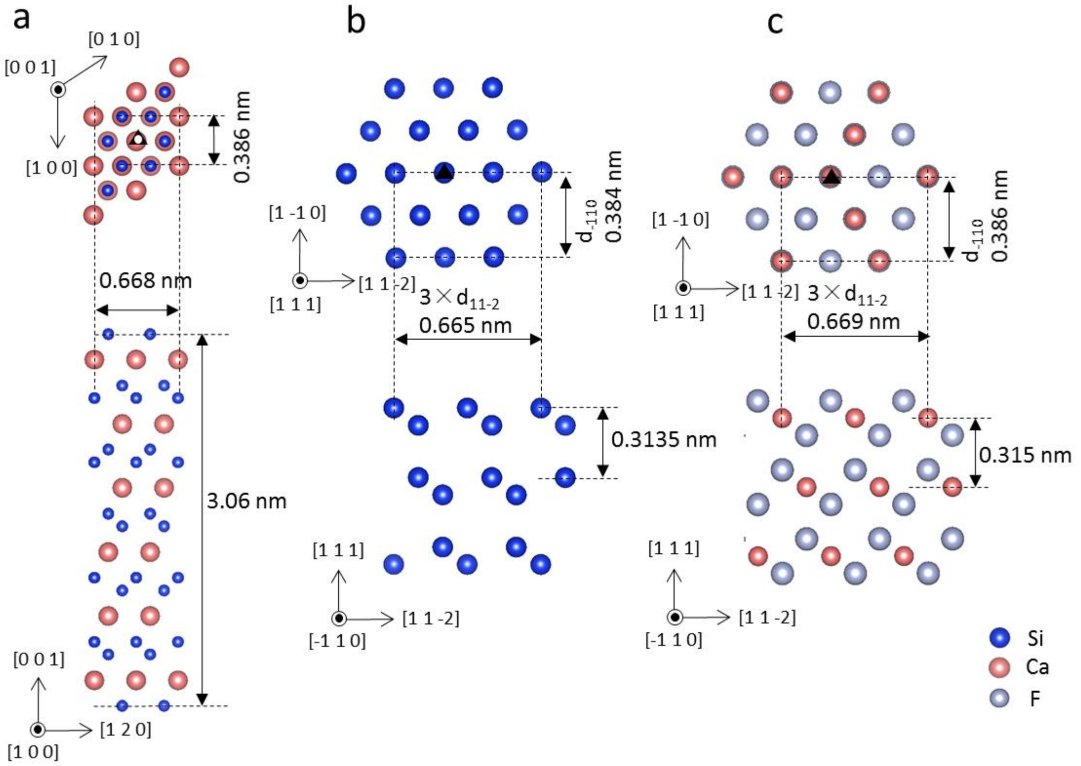

Supplementary Figure 9 | Models of $CaSi_2$ (**a**), cubic diamond Si (**b**) and CaF_2 (**c**).

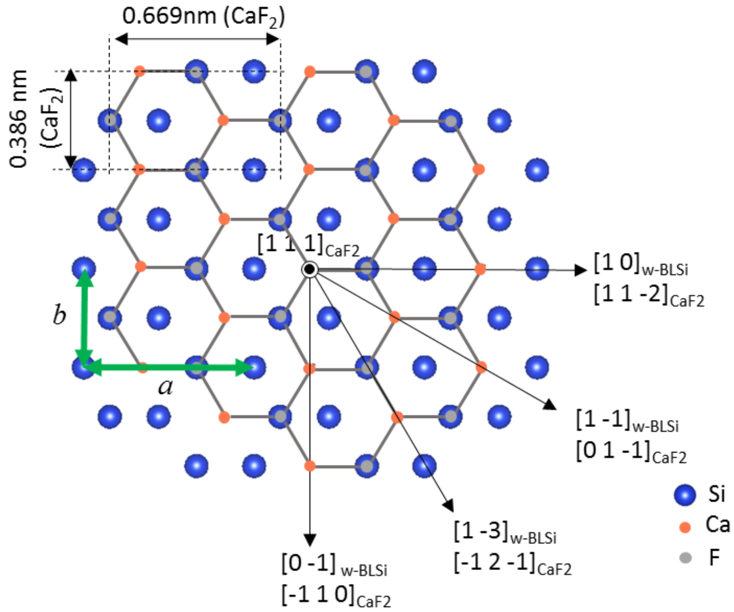

Supplementary Figure 10 | Atomic positions in the interface between the w-BLSi (001) plane and the CaF_2 (111) plane.

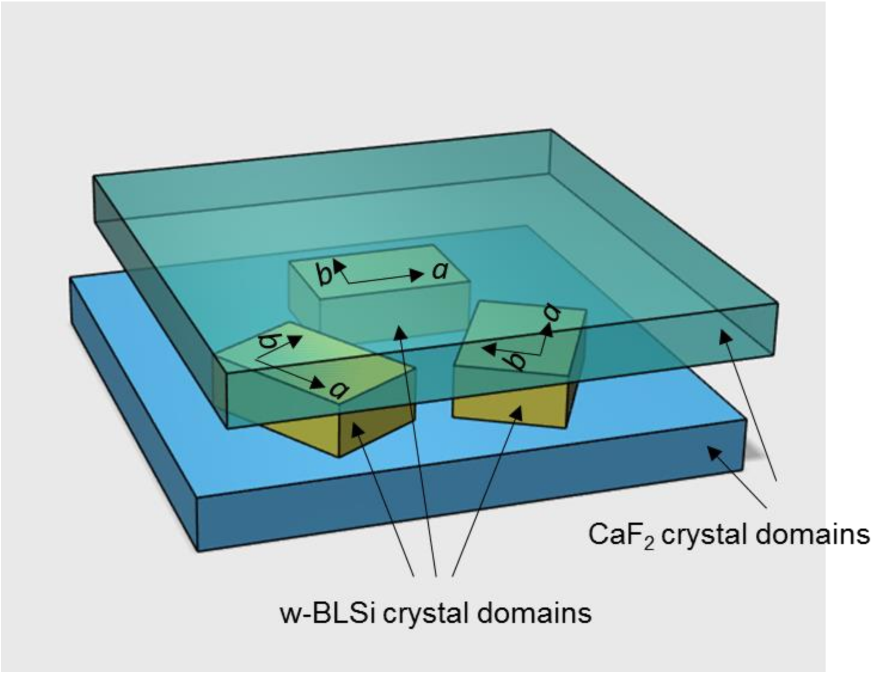

Supplementary Figure 11 | Three equivalent relative rotation angles between w-BLSi and the CaF_2 (111) plane.

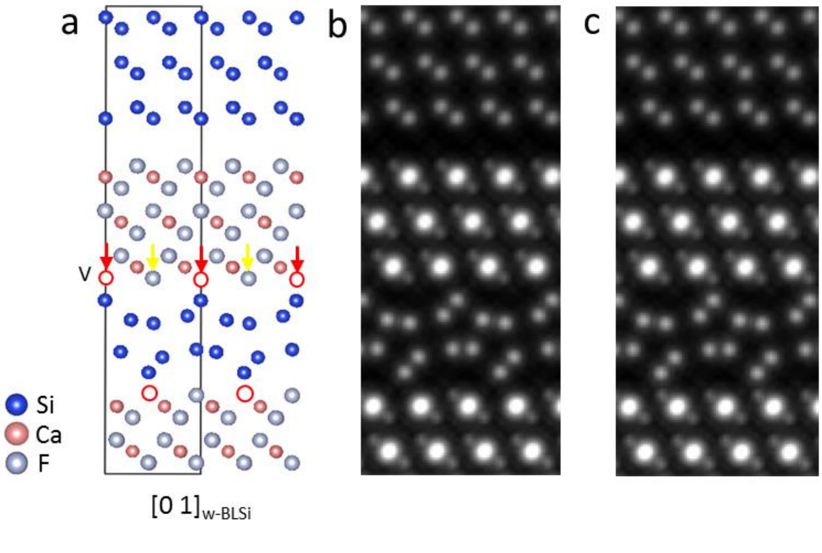

Supplementary Figure 12 | a, The long-period stacking structure model, which consists of bilayer CaF_2, w-BLSi, trilayer CaF_2 and Si based on the HAADF-STEM image (Supplementary Fig. 12a). F atoms (yellow arrows) and vacancies (red arrows) alternately substitute for F sites at the interface between w-BLSi and CaF_2. **b**, **c**, Simulation result with vacancies (**b**) and without vacancies (**c**) in the F site by MacTempasX. HAADF-STEM image simulations were demonstrated under the condition of 3.8 nm specimen thickness.

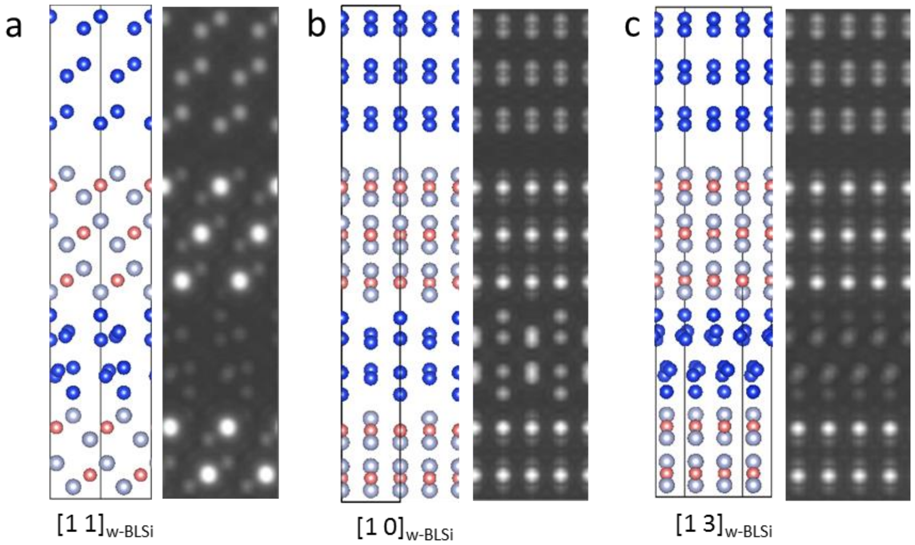

Supplementary Figure 13 | Projected model and simulated image of the Supplementary Fig. 14a unit cell along to the $[11]_{\text{w-BLSi}}$ direction (**a**), the $[10]_{\text{w-BLSi}}$ direction (**b**) and the $[13]_{\text{w-BLSi}}$ direction (**c**).

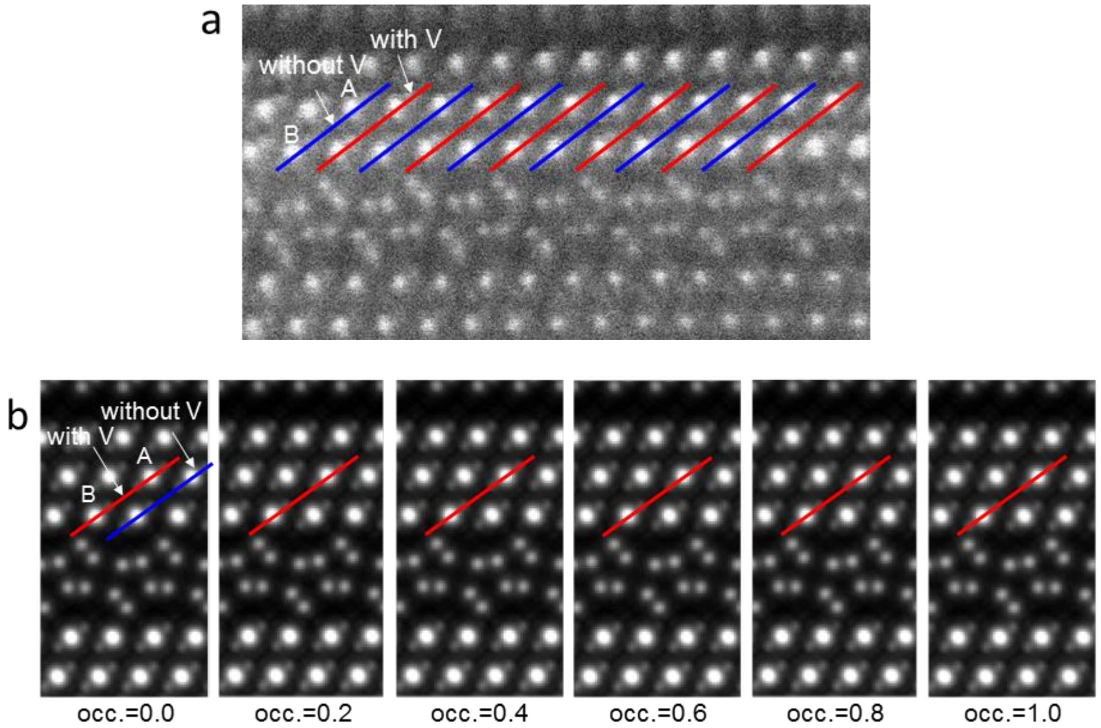

Supplementary Figure 14 | a, Observed HAADF-STEM image. **b**, HAADF-STEM simulation results for occupancies ranging from 0.0 to 1.0. Line profile positions with a vacancy and without a vacancy are indicated by red and blue lines, respectively.

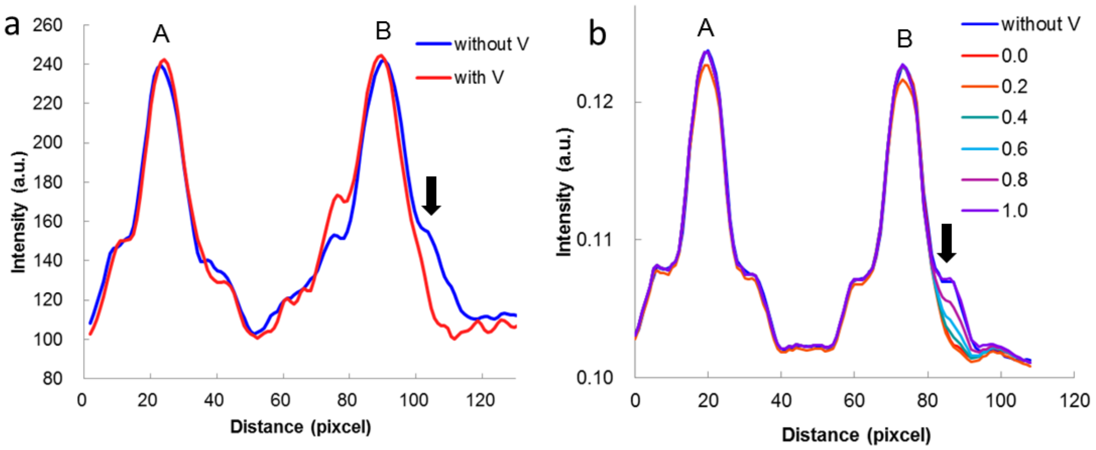

Supplementary Figure 15 | a, Average of line profiles with or without a vacancy in the observed HAADF-STEM image (Supplementary Fig. 14a). **b**, Line profiles of simulation results for occupancies ranging from 0.0 to 1.0 (Supplementary Fig. 14b).

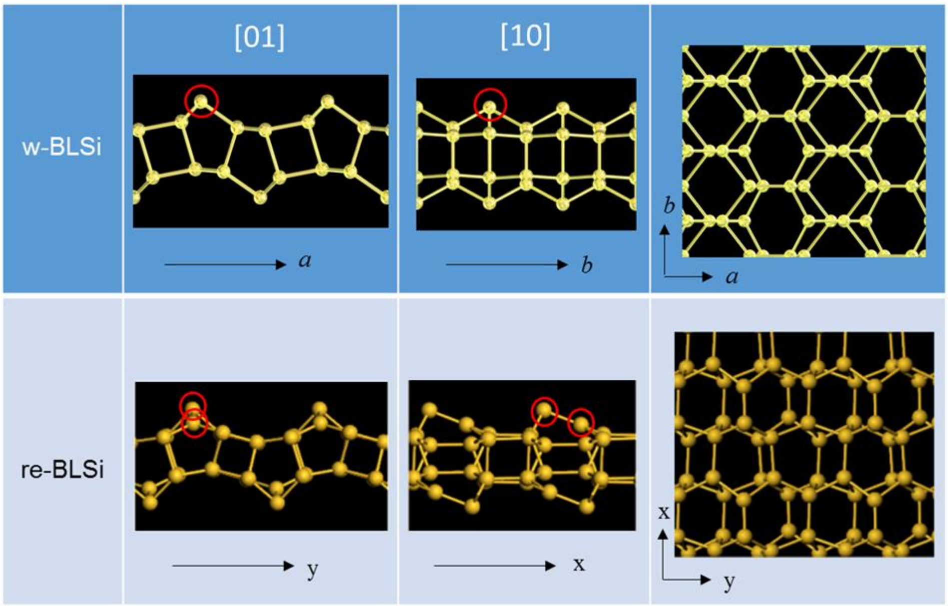

Supplementary Figure 16 | Comparison between w-BLSi and re-BLSi. The w-BLSi structure is from the experimental results of the present study. The re-BLSi structure is calculated from the results in Ref. 1. Although the predicted structure by MD calculation of re-BLSi[1] resembles w-BLSi obtained in the present study in the [01] direction, the image of w-BLSi differs from that of re-BLSi in the [10] direction. Because the experimental w-BLSi in the present study has higher symmetry on the b-axis than re-BLSi, the lattice constant of re-BLSi is the twice the period of that of w-BLSi on the b-axis.

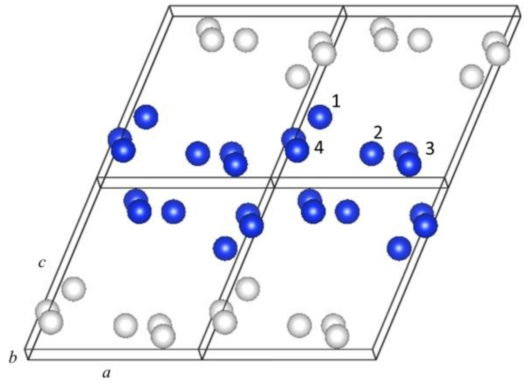

Supplementary Figure 17 | Schematic monoclinic 3D unit cell of the w-BLSi crystal of the *ab initio* MD result.

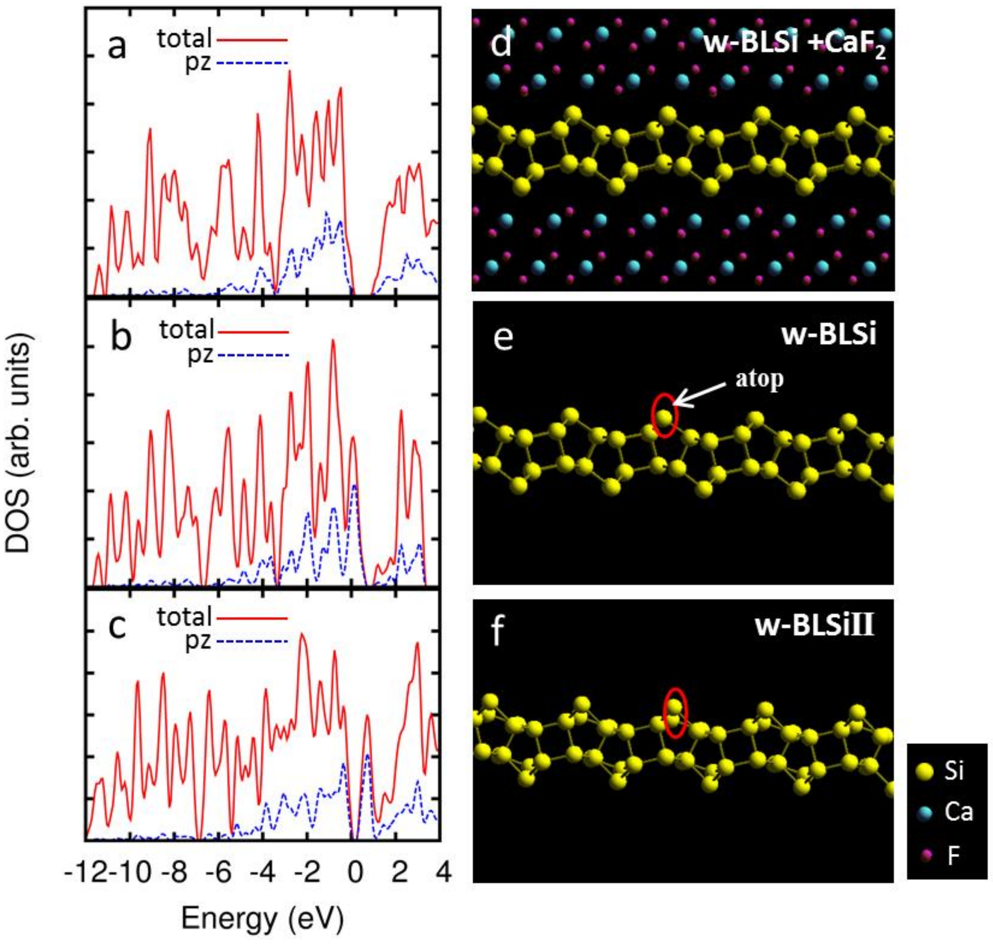

Supplementary Figure 18 | DOS for Si atoms in three structures of w-BLSi.

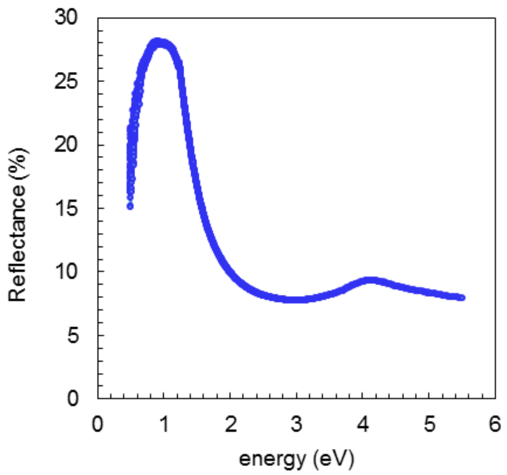

Supplementary Figure 19 | Diffuse reflectance spectrum as a function of energy for the $CaSi_2F_2$ compound.

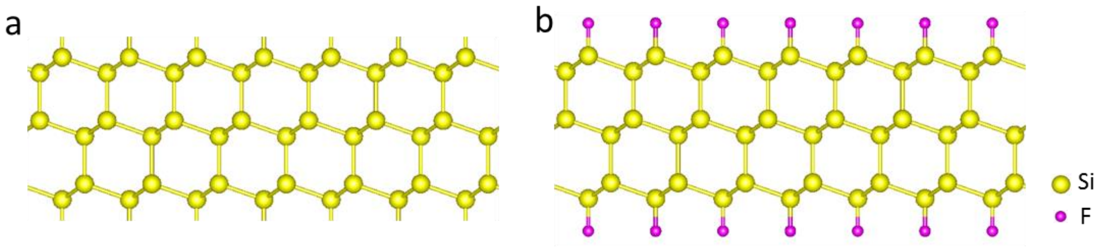

Supplementary Figure 20 | Trilayer silicene structure model. **a**, Trilayer silicene with dangling bonds. **b**, F atom-terminated trilayer silicene.

Supplementary Table 1 | Crystal structure parameters for the w-BLSi crystal regarded as a three-dimensional crystal.

Lattice parameter: a = 0.661(2) nm, b = 0.382(3) nm, c = 0.653(9) nm , α = β= γ= 90 °				
No.	x	y	z	Occ.
1	0.11(2)	0	0.67(1)	1
2	0.34(2)	0.5	0.65(1)	1
3	0.67(3)	0.5	0.71(3)	1
4	0.83(1)	0	0.88	1
5	0.00(1)	0	0.33(2)	1
6	0.29(1)	0	0.12(4)	1
7	0.46(2)	0.5	0.31(3)	1
8	0.79(2)	0.5	0.35(1)	1

Supplementary Table 2 | Crystal structure parameters for the w-BLSi crystal of MD calculated result.

Lattice parameter: a = 0.661 nm, b = 0.382 nm, c = 0.655 nm, α = β= γ= 90 °				
No.	x	y	z	Occ.
1	0.140(4)	0.999(3)	0.674(2)	1
2	0.344(2)	0.499(3)	0.637(5)	1
3	0.698(3)	0.501(5)	0.717(3)	1
4	0.837(5)	0.001(5)	0.881(3)	1
5	0.000(4)	0.999(4)	0.334(3)	1
6	0.293(3)	0.002(4)	0.115(4)	1
7	0.436(2)	0.502(4)	0.279(4)	1
8	0.794(3)	0.501(5)	0.362(2)	1

Supplementary Table 3 | Bond lengths of the w-BLSi crystal model of MD calculated result. (atom numbers given in Supplementary Fig. 12b)

	Bond lengths (nm)
Si1-Si2	0.235
Si2-Si3	0.240
Si3-Si4	0.238
Si1-Si5	0.241
Si2-Si7	0.242
Si3-Si8	0.241
Si5-Si6	0.241
Si6-Si7	0.239
Si7-Si8	0.243
Si8-Si5	0.235

Supplementary Table 4 | Bond angles of the w-BLSi crystal model of MD calculated result.

	Angle (degree)
Si1-Si2-Si1	109
Si1-Si2-Si3	122
Si4-Si3-Si4	107
Si1-Si2-Si7	104
Si3-Si2-Si7	88
Si2-Si3-Si8	93
Si4-Si3-Si8	110
Si1-Si5-Si6	104
Si5-Si6-Si7	93
Si5-Si6-Si7	95
Si6-Si7-Si6	106
Si2-Si7-Si6	110
Si2-Si7-Si8	92
Si3-Si8-Si7	88
Si3-Si8-Si5	103
Si5-Si8-Si5	109

Supplementary Table 5 | Crystal structure parameters for the w-BLSi crystal of MD calculation result regarded as a monoclinic 3D unit cell.

Lattice parameter：a = 0.661 nm, b = 0.382 nm, c = 0.710 nm, β = 67 °, space group *P*2/*m* (10)						
No.	Wyckoff pos.	Sym.	*x*	*y*	*z*	Occ.
1	2n	*m*	0.113(3)	0.5	0.382(3)	1
2	2n	*m*	0.500(3)	0.5	0.169(2)	1
3	2m	*m*	0.719(2)	0	0.137(3)	1
4	2m	*m*	0.040(2)	0	0.219(3)	1

Supplementary Note 1: Framework structure model of w-BLSi is consistent with the observed STEM images

HAADF-STEM images taken with the incident directions along the [1-10] and [11-2] directions of the CaF_2 crystal show complex contrast at the w-BLSi regions neighboring the CaF_2 layers (Supplementary Fig. 4). The w-BLSi layer is always sandwiched between CaF_2 thin-layered crystals (with thickness of 2, 3 or 4 layers). Supplementary Fig. 4a shows two different arrangements of bright dot contrasts in the w-BLSi regions, which correspond to the projection of atom columns of crystal along the electron incident directions. One arrangement shows a periodic sequence of two pentagons and a parallelogram, and the other arrangement shows a narrow hexagon and a parallelogram in the [1-10] incident direction of CaF_2. Furthermore, HAADF-STEM images taken with the [11-2] incident direction of CaF_2 also show two different arrangements of bright dot contrasts in the w-BLSi regions (Supplementary Figs. 4b and c). Four types of atom column arrangements can be recognised in the w-BLSi regions.

Assuming that the four types of atom column arrangements correspond to projections along the four different directions of the same crystal consisting of Si atoms only, we constructed a unique model of a Si atom network (framework structure), as shown in Fig. 2d, by trial and error. Although we considered several frameworks, the model shown in Fig. 2d is the only one that corresponds to the atom column arrangements of the HAADF-STEM images. Supplementary Fig. 8 shows the schematic drawings of the projected framework structure along several directions. The framework model is a rectangular 2D crystal with a 2D space group of *pm* and lattice parameters *a* close to the triple lattice spacing of d_{112} of CaF_2 (ca. 0.66 nm) and *b* close to d_{110} of CaF_2 (ca. 0.38 nm) (Supplementary Fig. 9). According to the 2D lattice of the framework model, the four directions in Supplementary Fig. 8 can be assigned as [10], [11], [13] and [01], respectively. It should be noted that the angle between the [10] and [13] directions is almost 60° and that the angle between [01] and [11] is also 60 °.

Supplementary Note 2: Atomic positions of the w-BLSi crystal determined by HAADF-STEM images

Because w-BLSi 2D crystals exist as one multi-phase in a crystallite, the lattice constants and atomic positions of w-BLSi could not be characterised by X-ray diffraction. In general, a HAADF-STEM image at high magnification with atomic resolution often suffers distortion due to specimen drift during the scan time. Thus, we determined the structure of w-BLSi by high-resolution transmission electron

microscopy (HRTEM) and HAADF-STEM images as accurately as possible. HAADF-STEM and HRTEM images taken of the CaF_2 region were used as standard images for calibrating the magnification and distortion.

The 2D lattice constants of w-BLSi were determined as a = 0.661(2) nm and b = 0.382(3) nm from the HRTEM images. Lattice constants were determined from the flat regions of w-BLSi (red arrows) in Supplementary Fig. 6, following calibrated magnification of an HRTEM image taken from the raw material of $CaSi_2$, with an estimated error of less than 1%. The projected atom positions of w-BLSi along the electron incident direction were measured from the bright dot contrast in the HAADF-STEM images with correcting image distortions by referring to the atom column positions in the nearest neighbor CaF_2 crystal regions.

Assuming that the thickness of w-BLSi (a distance from the center plane between the top Si of the five-membered ring and the F atomic plane to the other one, shown by black arrows in Supplementary Fig. 7a) is regarded to be virtually periodic in the third dimension, we could describe the 3D crystal structure as shown in Supplementary Table 1.

Supplementary Note 3: HAADF-STEM simulation

HAADF-STEM image simulations were performed using the structural parameters of the *ab initio* MD result in Supplementary Table 5. The simulated images of w-BLSi with the incident directions along the [01], [11], [10] and [13] directions are shown in Supplementary Figs. 4a to c, respectively. The contrasts in the simulated images show good agreement with those of the observed images.

By using long-period stacking structure models (Supplementary Fig. 12a) the same as the stacking sequence in Supplementary Fig. 7a, HAADF-STEM image simulations were performed to confirm the relative position between w-BLSi and the CaF_2 crystals as well as the F site occupancy on the CaF_2 (111) surface at the interface. As shown in Supplementary Fig. 12b, the simulated image calculated by the model with vacancies in half of the F sites shows better agreement with the observed contrast than that without vacancies (Supplementary Fig. 12c). In addition, Supplementary Figs. 13a to c show HAADF-STEM simulation images of the long-period stacking structure model (Supplementary Fig. 12a) in the $[11]_{\text{w-BLSi}}$, $[10]_{\text{w-BLSi}}$ and $[13]_{\text{w-BLSi}}$ directions. In each image, contrasts of Si, CaF_2, and w-BLSi agree with those in the observed images (Supplementary Figs. 4a to c).

HAADF-STEM image simulations were performed to confirm the occupancy of F

atoms at the vacancy site (red arrow in Supplementary Fig. 12a). Supplementary Fig. 14 shows observed and simulated HAADF-STEM images for occupancies ranging from 0.0 to 1.0. Line profiles obtained from the observed and simulated images are shown in Supplementary Fig. 15. The former was obtained from an average of the line profiles with or without a vacancy (Supplementary Fig. 14a), whereas the latter is the simulation result obtained by varying the occupancy from 0.0 to 1.0 (Supplementary Fig. 14b). The occupancy of F atoms at the vacancy site can be estimated as less than 0.4 by comparing the observed and simulated profiles (black arrows in Supplementary Fig. 15).

Supplementary Note 4: Atomic positions of the w-BLSi crystal determined by the *ab initio* MD result

Average atomic positions of the w-BLSi structure was determined by the *ab initio* MD result as shown in Supplementary Fig. 18d, which is the structure obtained in the quenching process at 0 K in the *ab* initio MD simulation. The experimentally determined lattice parameters a and b were employed in the *ab* initio MD run. Supplementary Table 2 shows atomic positions of the calculated w-BLSi crystal. The calculated-BLSi structure shows good agreement with the experimentally observed w-BLSi structure in Supplementary Table 1. Bond lengths and angles of the w-BLSi crystal of the MD calculated result model (atom numbers given in Supplementary Fig. 7b) are shown in Supplementary Tables 3 and 4, respectively.

The w-BLSi framework structure seems to have some symmetry elements including a mirror plane perpendicular to the *b*-axis, an inversion center at the center of the four-membered ring, and a twofold axis running through the center of the four-membered ring parallel to the *b*-axis. We could define a monoclinic 3D unit cell with a = 0.661 nm, b = 0.382 nm, c = 0.710 nm, and β = 67 ° and a space group of $P2/m$ (no. 10) in Supplementary Fig. 17. Supplementary Table 5 shows the lattice constants, space group and atomic positions of the w-BLSi crystal of the *ab initio* MD result.

Supplementary Note 5: Optical properties of w-BLSi

The main phases of the $CaSi_2F_{1.8\text{-}2.3}$ compound sample for diffuse reflectivity measurement are w-BLSi, trilayer silicene and the CaF_2 layer, which are two-dimensional crystals. Because the band gap opens from the calculation result of w-BLSi, we assume that the band dispersion approximates the parabolic model in near band edge region. The density of states for the two-dimensional crystal is constant as a

function of energy (D(E) = const) (three-dimensional crystal: (D(E) $\cong$ $E^{1/2}$). Therefore, the relationship between the absorption coefficient and the band gap energy can be described by the equations αhν = const (direct gap) and αhν = A (hν - Eg) (indirect gap), where α, h, ν, A and Eg are the absorption coefficient, Planck's constant, light frequency, proportional constant and band gap, respectively[2-5]. In the case of the three-dimensional crystal, $(\alpha h\nu)^n = A (h\nu - Eg)$, where n=1/2 for a direct transition and n=2 for an indirect transition. Then, the absorption coefficient of an indirect gap is proportional to the energy, whereas that of a direct gap is constant. The diffuse reflectance spectrum (Supplementary Fig. 19) is converted to the Kubelka-Munk function (K/S), which is proportional to the absorption coefficient, as shown in Fig. 3c.

The composition change from $CaSi_2$ to $CaSi_2F_X$ by F diffusion shows a primarily discontinuous increase and indicates several constant composition regions ($CaSi_2F_{1.5\text{-}1.8}$, $CaSi_2F_{2.0}$, and $CaSi_2F_{2.3}$), as shown in Supplementary Fig. 2c. Therefore, the $CaSi_2F_X$ composition and structure would immediately change upon F diffusion. When $CaSi_2F_X$ consists of only one type of Si structure, the relationship between the theoretical composition values and the layered compound structures is indicated as follows: $CaSi_2F_{1.5}$, w-BLSi and CaF_2 with F vacancy (occ. 0.5); $CaSi_2F_2$, trilayer silicene with dangling bonds and CaF_2; $CaSi_2F_{2.33}$, F-terminated trilayer silicene and CaF_2. Therefore, it is expected that there are two types of trilayer silicenes in the $CaSi_2F_{1.8\text{-}2.3}$ compound sample: one having unsaturated silicon bonds (dangling bonds) in $CaSi_2F_2$ and the other terminated with F atoms in $CaSi_2F_{2.3}$, as shown in Supplementary Fig. 20.

Supplementary Discussion: Electronic properties of w-BLSi

The DOS for Si atoms in the three w-BLSi systems are shown together with the decomposed DOS for p_z electrons in Supplementary Fig. 18 (the energy is measured from the Fermi level or the highest occupied level). Panel (a) shows the DOS for w-BLSi inside the CaF_2 crystal with an F-site surface vacancy of 0.5 [displayed in (d)], whereas panel (b) shows the DOS for w-BLSi under vacuum [displayed in (e)] with the same atomic configuration as in the CaF_2 crystal. Panel (c) shows the DOS for w-BLSi under vacuum [displayed in (f)], which was obtained by relaxing all of the Si atoms under vacuum while keeping the lattice constants used in (d) and (e). An up-down atomic arrangement [denoted by the red circle in (f)] in the row of the "atop" atoms is formed in the geometry optimization process, which lowers the energy by 0.02 eV/atom under vacuum (we call this structure "w-BLSiII" [see (f)]). By contrast, we found that w-BLSiII in the CaF_2 crystal is unstable when the CaF_2/Si interfaces have F-site

vacancies of 0.5 as in (d), which is consistent with the present experimental observations. Therefore, the surface morphology of w-BLSi can change depending on the details of the interfaces.

The key to understanding the surface morphology of w-BLSi is the presence of the dangling bonds on the “atop” atoms and the charge transfer between Si and Ca in the CaF_2 crystal. The total DOS for w-BLSi in a vacuum [see (b)] shows a half-filled band, which is mainly dominated by the p_z electrons from the dangling bonds on the “atop” atoms. Peierls instability is often induced in such a case; thus, the up-down arrangement of the “atop” atoms is formed, being seen as a Peierls distortion (w-BLSiII)[6]. The half-filled band in the DOS for w-BLSi [see (b)] is indeed split in the DOS for w-BLSiII [see (c)] with a gap of ~0.2 eV, resulting in lower energy than that of w-BLSi [unrelaxed structure: (e)] in a vacuum.

Moreover, the dangling bonds on the “atop” atoms in w-BLSi inside the CaF_2 crystal are partially saturated by the electrons transferred from the Ca atoms. In particular, fully saturated dangling bonds are expected when w-BLSi is sandwiched by the CaF_2 surfaces with an F-site vacancy of 0.5 [see (d)]. This can be confirmed by the DOS for w-BLSi in the CaF_2 crystal [see (a)], which shows no dangling-bond derived bands and a clear gap of 0.65 eV. Furthermore, we confirmed that w-BLSiII [see (f)] becomes unstable and is transformed to w-BLSi [see (d)] when w-BLSiII is sandwiched by the CaF_2 surfaces with an F-site vacancy of 0.5 by performing additional *ab initio* MD simulations. We thus conclude that the morphology and electronic properties of Si layers strongly depend on the nature of the substances into which they are intercalated.

Supplementary Method

Synthesis of $CaSi_2F_X$ compound. The $CaSi_2$ crystal structure has a two-dimensional Si network (silicene) equivalent to a buckled honeycomb Si (111) plane of bulk Si (Supplementary Fig. 1c). There are two types of $CaSi_2$ phases with different stacking sequences at ambient pressure: tr6-$CaSi_2$ (a = 0.3855 nm, c = 3.062 nm)[7] and tr3-$CaSi_2$ (a = 0.3829 nm, c = 1.590 nm)[8]. The tr6-$CaSi_2$ single-phase crystal was used as a starting material in the present study (Supplementary Fig. 1b). The $CaSi_2$ single crystals were synthesised by melting and solidifying 99.999 wt% Si and 99.99 wt% Ca with $CaSi_2$ stoichiometric composition in a Ta crucible under an argon atmosphere[9].

$CaSi_2$ single crystal grains (0.1 g) were reacted with 5 ml of ionic liquid [BMIM][BF_4] (1-butyl-3-methylimidazolium tetrafluoroborate) at 300 °C for 15 h. BF_4^- decomposed into F^- during annealing, and the $CaSi_2$ crystal was changed to $CaSi_2F_X$ compounds (0

≦ x ≦ 2.3) through the diffusion of F^-.

Computational method. Density functional theory (DFT) and *ab initio* molecular dynamics (MD) calculations were performed to calculate the density of states (DOS) and to examine the structural stability of BLSi using the Vienna *Ab initio* Simulation Package (VASP)[10]. The projector augmented wave method[11] and generalised gradient approximation (GGA) with the exchange and correlation functions of Perdew, Burke, and Ernzerhof (PBE) were employed[12]. A plane wave basis set with an energy cutoff of 400 eV was used with Γ-point sampling in the Brillouin zone. The supercell contained 96 Ca atoms, 64 Si atoms, and 192 (or 176) F atoms, with a lateral dimension of 1.3224 nm (x-direction) × 1.5296 nm (y-direction), which is consistent with the experimentally measured periodicity. To model the BLSi systems observed in our experiments, two-layer Si structures were sandwiched by CaF_2 crystal domains, each consisting of three sets of CaF_2 layers, with or without the F-site vacancy at the Si/CaF_2 interfaces.

The stability of the w-BLSi structure was examined by simulating the structural transformation of i-BLSi using *ab initio* MD simulations. The simulation was started with the i-BLSi structure sandwiched by CaF_2 domains with a slightly expanded space for i-BLSi (compared to the experimentally observed space) to facilitate the transformation to w-BLSi within the *ab initio* MD runs. The expanded space was used because it appeared to take significantly longer simulation time to complete the transformation with narrower spaces, which is not feasible with the present *ab initio* MD simulation.

The transformation immediately took place when the simulation was started with initial atomic velocities at 300 K. The upper and lower Si layers began to slide in opposite directions to each other, and some of the Si atoms (in the upper layer) were pushed down or up (in the lower layer), which resulted in a w-BLSi-like structure having four- and five-membered rings (note that the F vacancy was not included in this transformation simulation). Once the transformation took place, the dimensions of the space for the BLSi were readjusted to match the experimentally measured dimensions, and the F vacancy of 0.5 was introduced. The system was then equilibrated, and the resultant BLSi structure was found to perfectly agree with the experimentally observed w-BLSi structure. The 300 K first principles (FP) MD run was performed for ~1 ps following the equilibration process, confirming the stability of the w-BLSi structure. The DOS for the w-BLSi was calculated for the structure obtained in the quenching process (shown in Supplementary Fig. 18d) following the 300 K run.

Optical reflectivity. Diffuse reflectance spectra were obtained for the $CaSi_2F_{1.8\text{-}2.3}$ composition powder sample using a spectrophotometer (JASCO V-670). The $CaSi_2F_{1.8\text{-}2.3}$ compound was synthesised by annealing $CaSi_2$ single-crystal grains (average grain diameter of 1 mm) in ionic liquid [BMIM][BF_4] at 300 °C for 26 h. The $CaSi_2F_{1.8\text{-}2.3}$ powder was obtained by milling. Barium sulphate ($BaSO_4$) was used as the standard. The diffuse reflectance spectra were processed under the Kubelka-Munk formalism, and the band gaps were determined using a plot of the multiplication of the Kubelka-Munk function and energy.

Supplementary References:

1. Morishita, T., Spencer, M. J. S., Russo, S. P., Snook, I. K. & Mikami, M. Surface reconstruction of ultrathin silicon nanosheets. *Chem. Phys. Lett.* **506**, 221-225 (2011).
2. Lee, P. A., Said, G., Davis, R. & Lim, T. H. On the optical properties of some layer compounds. *J. Phys. Chem. Solids* **30**, 2719–2729 (1969).
3. Mak, K. F., Lee, C., Hone, J., Shan, J. & Heinz, T. F. Atomically thin MoS_2: A new direct-gap semiconductor. *Phys. Rev.Lett.* **105**, 136805 (2010).
4. Gaiser, C., et al. Band-gap engineering with HfS_XSe_{2-X}. *Phys. Rev. B* **69**, 075205 (2004).
5. Bianco, E., et al. Stability and exfoliation of germanane: A germanium graphane analogue. *ACS Nano* **7** 4414-4421 (2013).
6. Peierls, R. *Qua39ntum Theory of Solids* (Pergamon, Oxford, 1955).
7. Böhm, J. & Hassel, O. Die Kristallstuktur des Calciumsilicids $CaSi_2$. *Z. Anorg. Allg. Chem*. **160**, 152-164 (1927).
8. Dick, S. & Öhlinger, G. Crystal structure of calciumdisilicide, 3R-$CaSi_2$. *Z. Krist. New Cryst. Struct.* **213**, 232 (1998).
9. Yaokawa, R., Nakano, H. & Ohashi, M. Growth of $CaSi_2$ single phase polycrystalline ingots using the phase relationship between $CaSi_2$ and associated phases. *Acta Mater.* **81**, 41-49 (2014).
10. Kresse, G. & Furthmüller, J. Efficiency of ab-initio total energy calculations for metals and semiconductors using a plane-wave basis set. *Comput. Mater. Sci*. **6**, 15-50 (1996).
11. Blöchl, P. E. Projector augmented-wave method. *Phys. Rev. B* **50**, 17953 (1994).
12. Perdew, J. P., Burke, K. & Ernzerhof, M. Generalized Gradient Approximation

Made Simple. *Phys. Rev. Lett*. **77**, 3865 (1996).
